# Anti-Inflammatory and Immunomodulatory Effects of Aqueous Extracts from Green Leaves and Rhizomes of *Posidonia oceanica* (L.) Delile on LPS-Stimulated RAW 264.7 Macrophages

**DOI:** 10.3390/molecules30244685

**Published:** 2025-12-07

**Authors:** Giulia Abruscato, Daniela Ganci, Federica Bellistrì, Roberto Chiarelli, Manuela Mauro, Aiti Vizzini, Vincenzo Arizza, Mirella Vazzana, Claudio Luparello

**Affiliations:** 1 Dipartimento di Scienze e Tecnologie Biologiche Chimiche e Farmaceutiche (STEBICEF), Università di Palermo, 90128 Palermo, Italy; giulia.abruscato@unipa.it (G.A.); daniela.ganci01@unipa.it (D.G.); federica.bellistri@community.unipa.it (F.B.); roberto.chiarelli@unipa.it (R.C.); manuela.mauro01@unipa.it (M.M.); aiti.vizzini@unipa.it (A.V.); vincenzo.arizza@unipa.it (V.A.); mirella.vazzana@unipa.it (M.V.); 2 National Biodiversity Future Center (NBFC), 90133 Palermo, Italy

**Keywords:** Nos2, COX-2, TNFα, IL-1β, IL-6, IL-10, NF-κB, MAPK, AKT, endocytosis

## Abstract

The marine angiosperm *Posidonia oceanica* (Linnaeus) Delile, 1813 is a rich source of phytotherapeutic compounds whose potential applications for human health remain largely uninvestigated. Here, we determined the differential impact of aqueous extracts from *P. oceanica*’s green leaves (GLE) and rhizomes (RE) on the inflammation-related mRNA expressions and protein levels, nitric oxide (NO) release, and endocytic activity in LPS-stimulated RAW 264.7 macrophages. We also examined the influence of the extracts in modulating the activation of components of intracellular signaling pathways. Co-treatments of LPS-stimulated RAW 264.7 cells in the presence of either GLE or RE resulted in a reduction in NO production, associated with a down-regulation of *Nos2* expression, reduced levels of COX-2 and TNFα proteins, and a decrease in *Nfkb1* expression and NF-κB activation. No effect was exerted on the release of IL-6. Moreover, co-exposures to LPS and the extracts led to an elevation in pJNK and pAKT levels alongside a reduction in pERK. In contrast to GLE, RE specifically lowered IL-1β production, induced a more robust increase in IL-10, positively influenced the endocytic function of RAW 264.7 cells, and drastically up-regulated the phosphorylation of p38. The data obtained indicate that GLE and RE exhibit considerable promise as prospective anti-inflammatory and immunomodulatory agents.

## 1. Introduction

*Posidonia oceanica* (L.) Delile, 1813 (Liliopsida, Alismatales: Posidoniaceae) is a Mediterranean endemic plant that develops extensive underwater meadows. This angiosperm is instrumental in ecosystem engineering, providing habitat and refuge for a range of organisms [1], and has been identified as effective indicators for monitoring both biotic and abiotic stressors [2]. From an anatomical perspective, *P. oceanica* features long, ribbon-shaped foliage, rhizomes that serve as altered stem structures, and brief roots, with each part displaying unique phytochemical characteristics [3,4,5].

Historically, coastal communities have utilized seagrass as a food source and as traditional herbal treatments for various health problems, such as skin and throat irritation, acne, and joint discomfort [6,7]. Recent studies have produced both in vitro and in vivo evidence that highlights the anti-tumor, anti-adipogenic, anti-diabetic, and antioxidant mechanisms associated with the extracts of *P. oceanica*, e.g., [8,9,10,11,12].

A wide range of natural substances and food products has been shown to influence immune system activity, acting as anti-inflammatory/immunoregulatory agents or immunostimulants that improve defense mechanisms by activating neutrophils and macrophages, e.g., [13,14,15]. These latter cells are crucial for triggering both anti- and pro-inflammatory responses. In particular, macrophages play three pivotal roles during inflammation, that is, antigen presentation, phagocytosis, and the production of an array of cytokines and growth factors [16]. Bacterial lipopolysaccharide (LPS) is one of the most widely used pro-inflammatory stimuli. At a molecular level, LPS promotes the activation of intracellular signaling which, in turn, induces the translocation of activated nuclear factor-kappa B (NF-κB) from the cytoplasm to the nuclear compartment. Upon activation of NF-κB, the genes encoding for the enzymes inducible nitric oxide synthase (iNOS) and cyclooxygenase 2 (COX-2) are up-regulated, resulting in the generation of the immune mediators nitric oxide (NO) and prostaglandins, respectively. These substances are released together with cytokines, all of which contribute to the progression of the immunological cascade [17]. Interestingly, by utilizing ethanolic extracts of *P. oceanica*’s leaves, Vasarri et al. [18] investigated their anti-inflammatory properties in LPS-treated RAW 264.7 murine macrophages and identified an antioxidant impact linked to the inhibition of intracellular signaling pathways and NF-κB activation.

Here, we assessed the effects of two additional matrices from *P. oceanica*, that is, the aqueous extracts from green leaves (GLE) and rhizomes (RE), on the modulation of the inflammatory signaling in LPS-stimulated RAW 264.7 cells vs. control. Our previous research revealed distinct differences in the polyphenolic and protein profiles of GLE and RE, both of which demonstrated cytotoxic effects on HepG2 liver cancer cells through independent death-inducing mechanisms [5]. Also, GLE alone has presented potential anti-diabetic properties in vitro [8]. Notably, in the context of inflammatory conditions, both extracts demonstrated protective and reparative properties in an in vitro model of the human blood–brain barrier exposed to TNFα, as evidenced by the decrease in nitric oxide (NO) production and NLRP3 inflammasome expression, alongside the restoration of the integrity of tight and adherens junctions [19]. Therefore, a combination of spectrophotometric assays, flow cytometry, quantitative reverse transcription-polymerase chain reaction (qRT-PCR), ELISA, and protein blotting techniques was employed to assess the differential impact of each extract on the expression of inflammation-related genes and proteins, NO release, and endocytic activity in macrophages. This study also examined the influence of the extracts in modulating the activation of components of intracellular signaling pathways. The impact of this research lies in elucidating how two different matrices of *P. oceanica*, namely the aqueous extracts from green leaves and rhizomes, distinctly modulate inflammatory signaling in macrophages. While not directly focused on immediate pharmaceutical product development, this research provides fundamental insights that could support future biomedical and nutraceutical applications. The results obtained show that these two extracts exert diversified influences on the key mechanisms underlying inflammation, expanding and reinforcing the established knowledge regarding the antioxidant, anti-diabetic, and cytotoxic properties attributed to this marine organism.

## 2. Results

### 2.1. Effects of GLE and RE on the Viability and NO Production in RAW 264.7 Cells Cultured in Control Conditions

**Figure 1:**
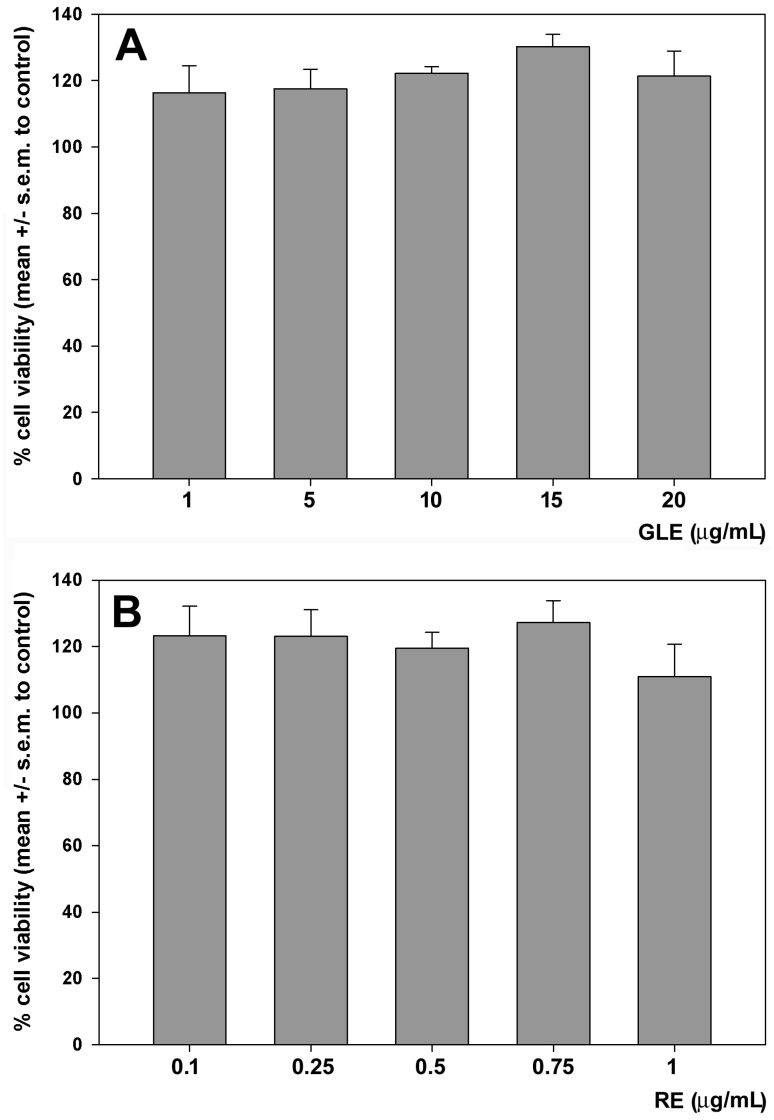
Dose–response effect of GLE (**A**) and RE (**B**) on the viability of RAW 264.7 cells after 24 h of exposure, quantified using the MTT assay. The error bars correspond to the standard error of the mean (s.e.m.) of three independent measurements. One-way ANOVA followed by Holm–Sidak comparison procedure was used. Normality test passed.

Before examining the potential impact of GLE and RE on LPS-stimulated RAW 264.7 cells, the individual extracts were evaluated for cytotoxic and inflammatory response using the MTT and Griess assays, respectively. As shown in Figure 1, the administration over 24 h of various concentrations of GLE (Figure 1A) and RE (Figure 1B), chosen due to their previously confirmed lack of cytotoxicity towards HepG2 cells [5], did not result in a decline in cell viability. Conversely, the results indicated a non-significant upward trend in viability ranging from 16% at 1 μg/mL GLE to 30% at 15 μg/mL GLE.

**Figure 2:**
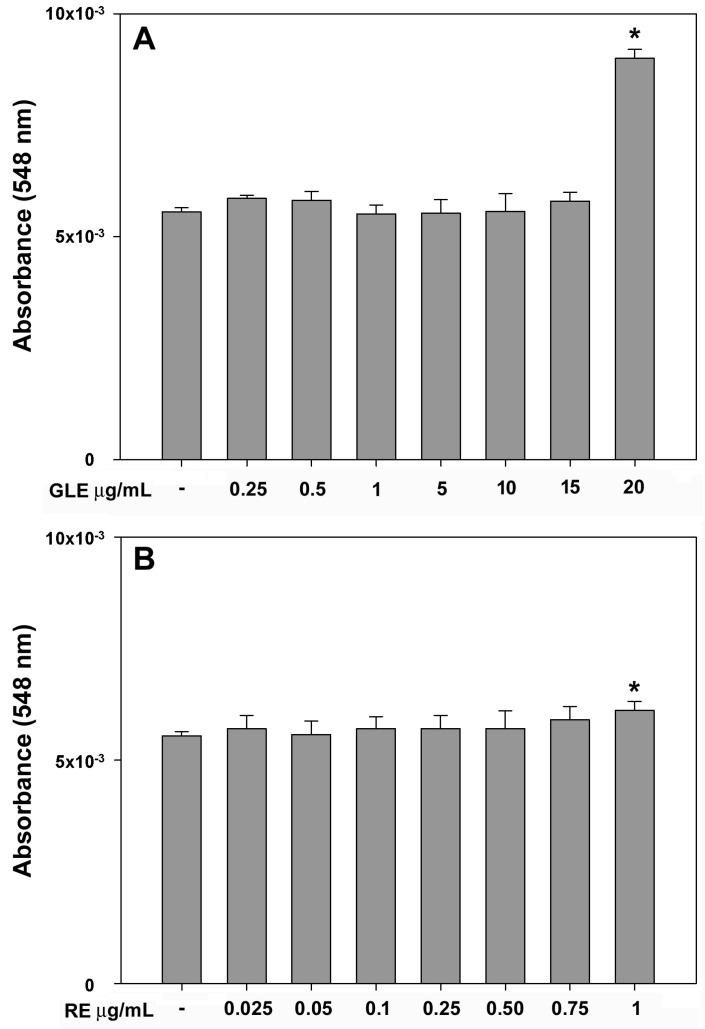
Dose–response effect of GLE (**A**) and RE (**B**) on NO release in the culture media of RAW 264.7 cells after 24 h of exposure, quantified using the Griess reaction. The error bars correspond to the standard error of the mean (s.e.m.) of three independent measurements. One-way ANOVA followed by Holm–Sidak comparison procedure was used. * *p* < 0.05 compared to control. Normality test passed.

A wider array of GLE and RE concentrations was tested on macrophages to explore the possible induction of NO release, which reflects a pro-inflammatory status in immune cells. As depicted in Figure 2, after a 24 h exposure to the extracts, no effects were observed across all the assayed concentrations, with the exception of the highest amount of GLE (i.e., 20 μg/mL), which led to a significant +62% increase (Figure 2A), and, to a lesser extent, RE (i.e.,1 μg/mL), which resulted in a significant +10% increase (Figure 2B).

### 2.2. Effects of GLE and RE on NO Production and iNOS mRNA Expression Level in LPS-Treated RAW 264.7 Cells

**Figure 3:**
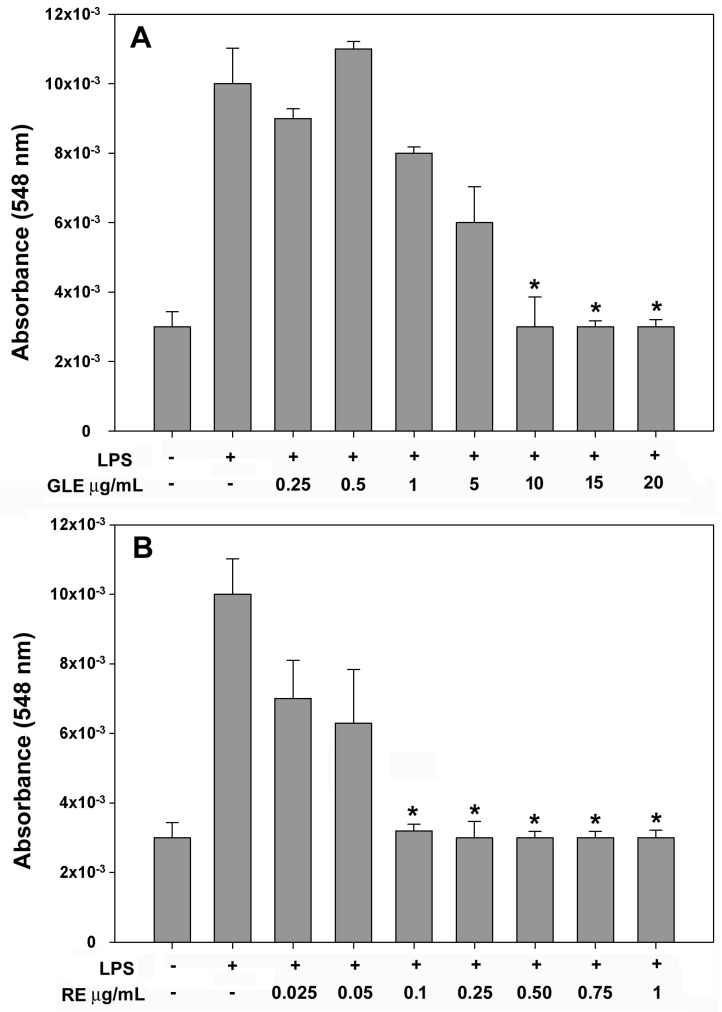
Dose–response effect of co-treatment with 0.1 μg/mL LPS and GLE (**A**) or RE (**B**) on NO release in the culture media of RAW 264.7 cells after 24 h of exposure, quantified using the Griess reaction. The error bars correspond to the standard error of the mean (s.e.m.) of three independent measurements. One-way ANOVA followed by Holm–Sidak comparison procedure was used. * *p* < 0.05 compared to LPS-treated cells. Normality test passed.

After determining that GLE and RE had no detrimental effect on macrophages’ viability and NO release, we examined whether the co-treatment with LPS and either extract at varying concentrations could attenuate NO production. Figure 3 shows that exposure to LPS markedly elevated NO release (about +3.3-fold vs. control), as expected. In line with the data reported in [19], the co-application of increasing amounts of either extract led to a significant reduction in NO production to levels akin to the control when GLE was administered at 10–20 μg/mL (Figure 3A) and RE at 0.1–1 μg/mL (Figure 3B). Based on these data, we chose 10 μg/mL GLE and 0.1 μg/mL RE, i.e., the lowest concentrations capable to fully counteract the LPS-induced NO up-regulation, for further investigations into some molecular aspects of the extracts’ anti-inflammatory and immunomodulatory activities.

To further validate the capacity of GLE and RE to inhibit NO production by LPS-stimulated macrophages, we investigated the extracts’ role in modulating iNOS mRNA expression. As shown in Table 1, the qRT-PCR analysis confirmed a significant enhancement in *Nos2* expression level following LPS stimulation of the cells. Conversely, the co-administration of 10 μg/mL GLE and, to a greater extent, 0.1 μg/mL RE significantly reduced the mRNA expression of iNOS to levels that were lower than those found in the control condition.

### 2.3. Effects of GLE and RE on Cyclooxygenase-2 (COX-2) mRNA Expression and Protein Levels in LPS-Treated RAW 264.7 Cells

COX-2, an inducible COX isoform encoded by the *Ptgs2* gene, serves as a crucial inflammation-associated enzyme, catalyzing the production of prostaglandin E2 from arachidonic acid in activated macrophages [20]. We examined the extracts’ role in modulating COX-2 transcription level in LPS-treated macrophages. As shown in Table 2, by analogy with *Nos2*, the administration of 10 μg/mL GLE, along with a more pronounced effect from 0.1 μg/mL RE, markedly decreased the LPS-stimulated *Ptgs2* expression to about 55% and 45%, respectively, both of which are below the control condition levels.

**Figure 4:**
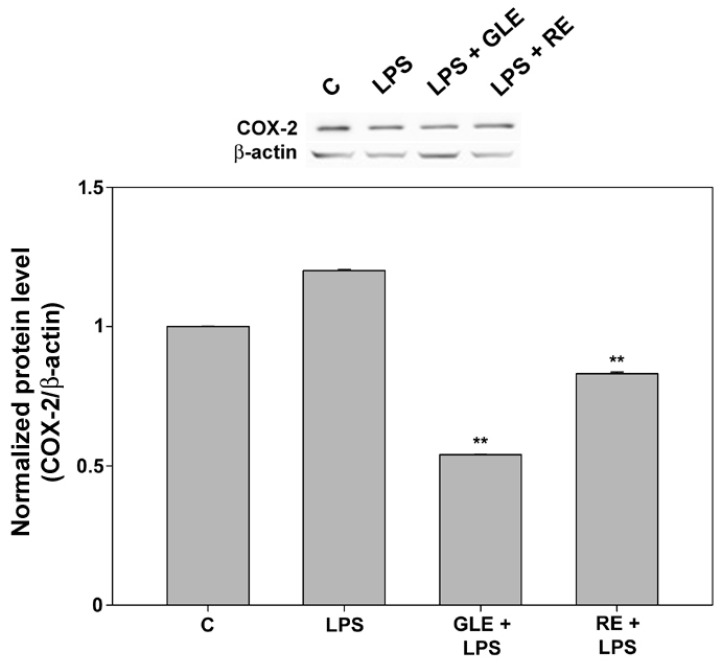
Effect of co-treatment with 0.1 μg/mL LPS and 10 μg/mL GLE or 0.1 μg/mL RE on COX-2 protein levels in RAW 264.7 cells, determined by Western blot. Each bar is representative of at least three independent experiments performed in triplicate. Values are expressed as mean fold change ± s.e.m. compared to the control (C). One-way ANOVA followed by Holm–Sidak comparison procedure was used. ** *p* < 0.001 vs. LPS. Normality test vs. control passed.

To confirm the gene expression results, the impact of co-treatment with LPS and each extract on COX-2 protein levels was assessed using Western blot analysis. As displayed in Figure 4, the co-exposure to RE and, more markedly, GLE counteracted the rise in the expression level induced by LPS alone, reducing it to about 69% and 45%, respectively, and to levels even below those seen in the control condition.

### 2.4. Effects of GLE and RE on mRNA Expression and Protein Levels of Inflammatory Biomarkers in LPS-Treated RAW 264.7 Cells

**Figure 5:**
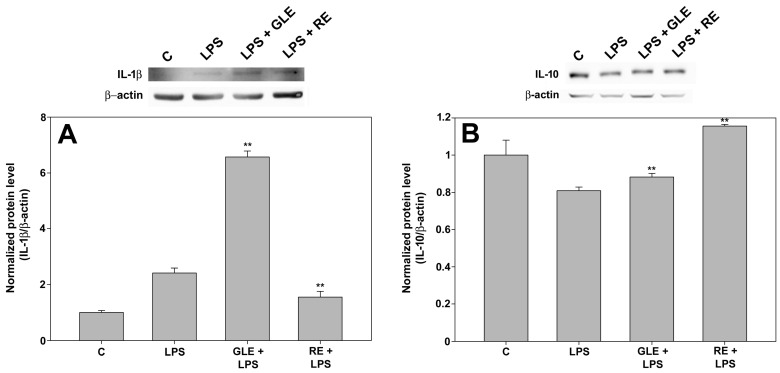
Effect of co-treatment with 0.1 μg/mL LPS and 10 μg/mL GLE or 0.1 μg/mL RE on IL-1β (**A**) and IL-10 (**B**) protein levels in RAW 264.7 cells, determined by Western blot. Each bar is representative of at least three independent experiments performed in triplicate. Values are expressed as mean fold change ± s.e.m. compared to the control (C). One-way ANOVA followed by Holm–Sidak comparison procedure was used. ** *p* < 0.001 vs. LPS. Normality test vs. control passed.

**Figure 6:**
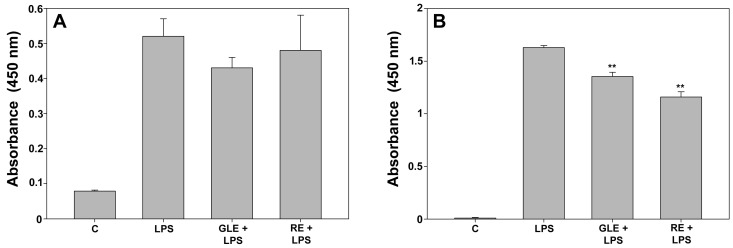
Effect of co-treatment with 0.1 μg/mL LPS and 10 μg/mL GLE or 0.1 μg/mL RE on the levels of IL-6 (**A**) and TNFα (**B**) secreted in the media of RAW 264.7 cells, determined by ELISA. Each bar is representative of at least three independent experiments performed in triplicate. Values are expressed as mean fold change ± s.e.m. compared to the control (C). One-way ANOVA followed by Holm–Sidak comparison procedure was used. ** *p* < 0.001 vs. LPS. Normality test vs. control passed.

It is recognized that activated macrophages release pro-inflammatory cytokines like TNFα, IL-1β, and IL-6, while IL-10 is mainly regarded as a cytokine-synthesis inhibitor that, once secreted, reduces and terminates immune reactions [21]. The effect of 10 μg/mL GLE and 0.1 μg/mL RE treatments on the production of inflammatory mediators by LPS-stimulated RAW 264.7 cells was studied by measuring the mRNA expression and protein levels of the mentioned cytokines via qRT-PCR (for IL-1β, IL-6, and IL-10, shown in Table 3), Western blot (for IL-1β and IL-10, shown in Figure 5), and ELISAs (for IL-6 and TNFα, shown in Figure 6). The experimental outcomes pointed to a quali-quantitatively diverse immunomodulatory effect of the extracts. In particular, co-exposure to LPS and GLE induced up-regulation of IL-1β mRNA (about +68%, Table 3) and protein (about +173%, Figure 5A) whereas RE triggered a reduction in IL-1β protein to approximately 36% (Figure 5A) in comparison to LPS-challenged cells. In addition, despite the modest but significant boost in IL-6 mRNA expression upon exposure to the extracts, IL-6 protein release into the medium nevertheless showed no change under both experimental conditions compared to LPS-treated cells (Figure 6A). On the other hand, GLE and RE co-treatments induced a slight reduction in TNFα amount in the medium, reaching about 83% and 71% compared to LPS-treated cells, respectively, (Figure 6B), although not matching the levels observed in the control condition. Concerning the anti-inflammatory cytokine IL-10, co-exposure to GLE elicited a minor rise in its protein level by about 9% compared to LPS-treated cells (Figure 5B); of note, the co-treatment with RE produced a prominent up-regulation of both IL-10 mRNA (Table 3) and protein (Figure 5B) by about 30% and 43%, respectively, compared to LPS-treated cells, with the resulting protein level slightly but significantly exceeding that found in the control condition.

### 2.5. Effects of GLE and RE on NF-κB p105 mRNA Expression Level and NF-κB Activation in LPS-Treated RAW 264.7 Cells

**Figure 7:**
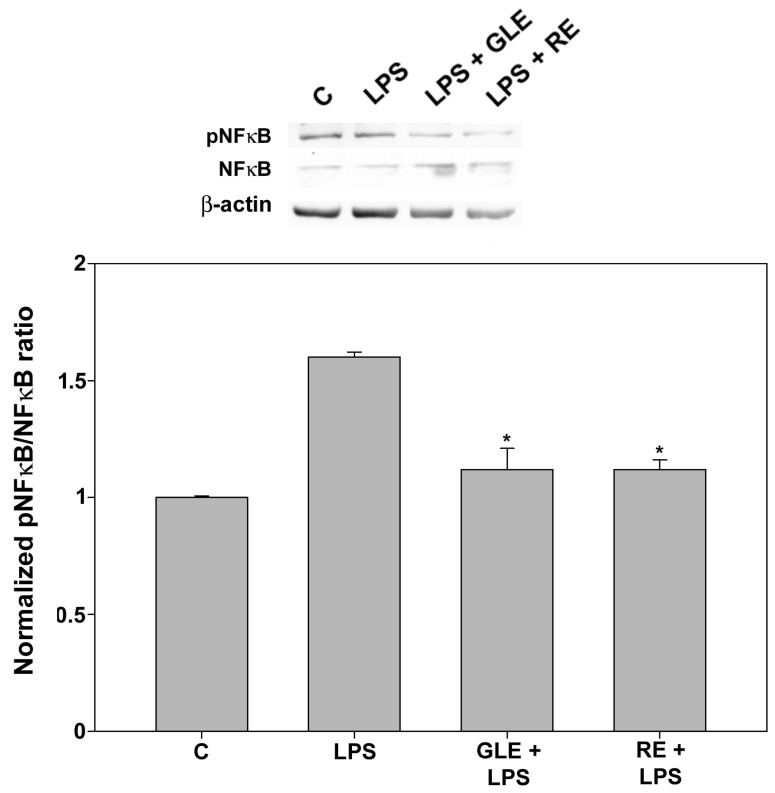
Effect of co-treatment with 0.1 μg/mL LPS and 10 μg/mL GLE or 0.1 μg/mL RE on NF-κB activation in RAW 264.7 cells, determined by Western blot. Each bar is representative of at least three independent experiments performed in triplicate. Values are expressed as mean fold change ± s.e.m. compared to the control (C). One-way ANOVA followed by Holm–Sidak comparison procedure was used. * *p* < 0.05 vs. LPS. Normality test vs. control passed.

The transcription factor NF-κB is a critical orchestrator of immune responses and inflammation upon activation of its target genes. When the p65 subunit of the NF-κB dimer is phosphorylated at Ser536 in the transactivation domain, its conformation is altered, enhancing its pro-inflammatory transcriptional activity [22]. We examined whether the extracts regulated the NF-κB signaling pathway in LPS-stimulated macrophages, quantifying the mRNA expression of NF-κB1 (p105) and the extent of the NF-κB1 p65 subunit’s activation by phosphorylation. As shown in Table 4 and Figure 7, treatment with both 10 μg/mL GLE and 0.1 μg/mL RE reduced the LPS-induced *Nfkb1* expression and the degree of p65 phosphorylation, achieving conditions comparable to the control.

### 2.6. Effects of GLE and RE on the Activation of Signaling Pathways in LPS-Treated RAW 264.7 Cells

**Figure 8:**
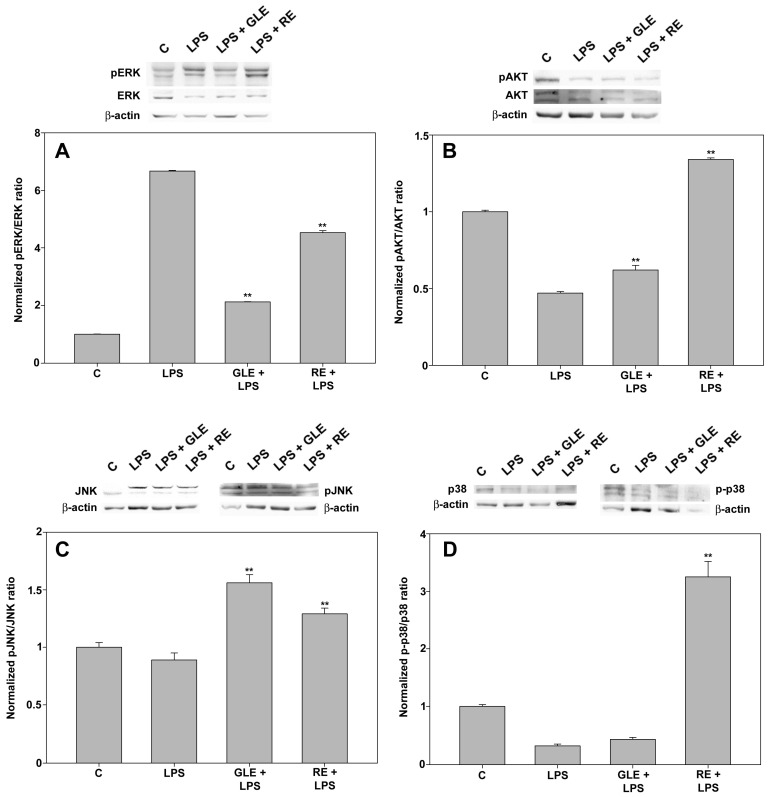
Effect of co-treatment with 0.1 μg/mL LPS and 10 μg/mL GLE or 0.1 μg/mL RE on the activation of ERK (**A**), AKT (**B**), JNK (**C**), and p38 (**D**) in RAW 264.7 cells, determined by Western blot. Each bar is representative of at least three independent experiments performed in triplicate. Values are expressed as mean fold change ± s.e.m. compared to the control (C). One-way ANOVA followed by Holm–Sidak comparison procedure was used. ** *p* < 0.001 vs. LPS. Normality test vs. control passed.

The potential role of MAPK (ERK, JNK, and p38) and AKT signaling pathways was further investigated to shed light on the molecular mechanism behind GLE- and RE-mediated regulation of immune mediators in LPS-stimulated macrophages. Under the established experimental setup, LPS elicited a robust activation of ERK and down-regulation of the pAKT/AKT and p-p38/p38 ratios, whereas no significant impact was detected on JNK phosphorylation. Also, in this scenario, co-exposure to the extracts yielded an effect divergent in its qualitative and quantitative characteristics. As depicted in Figure 8, the panel shows that co-treatment with GLE induced massive down-regulation of the pERK/ERK ratio (about −68%, Figure 8A), coupled with up-regulation of activated AKT (about +31%, Figure 8B) and, more notably, JNK (about +77%, Figure 8C), compared to the LPS-only condition. On the other hand, co-treatment with RE, while leading to less prominent down-regulation of activated ERK (about −32%, Figure 8A), prompted up-regulation of activated JNK (about +45%, Figure 8C), AKT and, far more pronouncedly, p38 (about 1.35-fold and 9-fold, respectively, shown in Figure 8B,D), exceeding the amounts found in the control condition.

### 2.7. Effect of GLE and RE on the Bulk-Phase Endocytic Acyivity of LPS-Treated RAW 264.7 Cells

**Figure 9:**
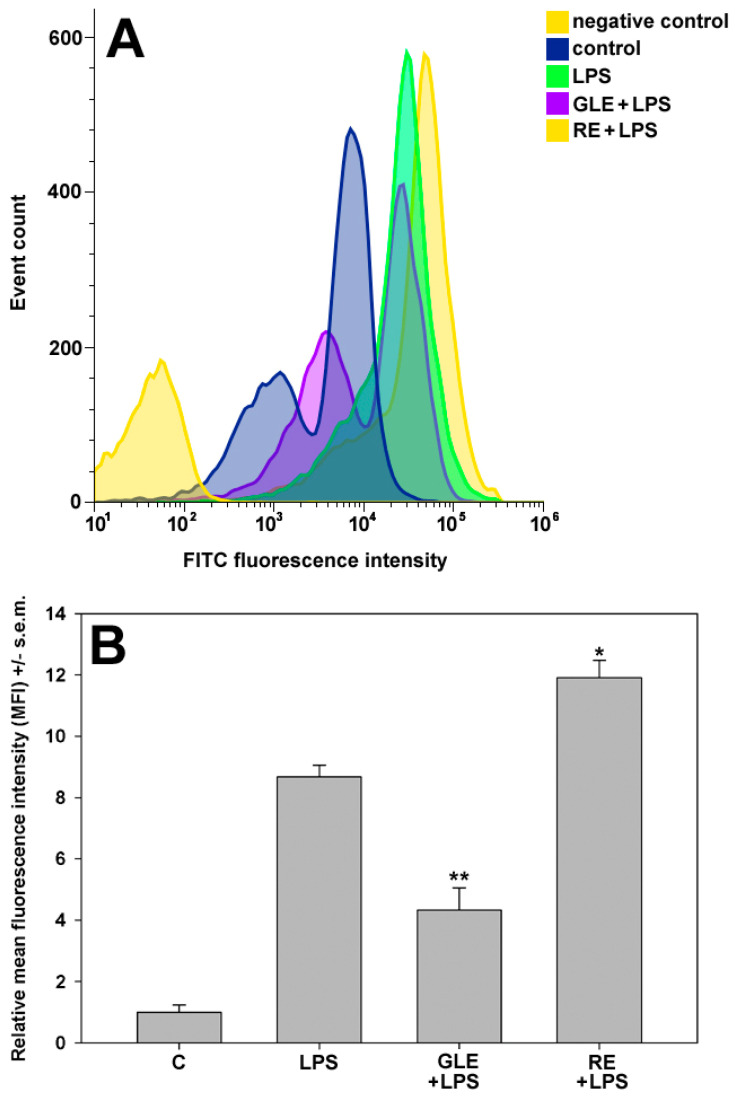
(**A**) Representative flow cytometric profiles of FITC-dextran uptake by RAW 264.7 cells cultured under control conditions, treated with 0.1 μg/mL LPS or co-treated with LPS and 10 μg/mL GLE or 0.1 μg/mL RE. Fully processed preparations without FITC-dextran were analyzed in parallel for the control of background derived from autofluorescence (negative control). (**B**) Bar graph showing the relative mean fluorescence intensity (MFI) of control (C), LPS, GLE + LPS, or RE + LPS-treated RAW 264.7 cells. All the results were analyzed for the MFI of each condition, and the MFI of either treated cell population was divided by the MFI of the controls to normalize the data. Error bars indicate the s.e.m. of three independent measurements. One-way ANOVA followed by Holm–Sidak comparison procedure was used. * *p* < 0.05 and ** *p* < 0.001 vs. LPS. Normality test vs. control passed.

The essential functions of macrophages include bulk-phase uptake, otherwise known as macropinocytosis, which plays a primary role in antigen presentation. In addition, through constitutive macropinocytosis solutes containing pathogen- and danger-associated molecular patterns are internalized and subsequently recognized by innate immune sensors within endomembranes or the cytosol [23]. To gain further insight into the potential regulatory involvement of GLE and RE in the endocytic activity of RAW 264.7 cells exposed to LPS, the uptake of FITC-dextran was assessed utilizing flow cytometry. Predictably, as displayed in Figure 9, the administration of LPS substantially enhanced the phagocytic capacity of RAW 264.7 cells (about +7.6-fold vs. control). On the other hand, co-exposure to the extracts produced a contrasting effect; compared to the LPS group, GLE reduced the macrophages’ ability for FITC-dextran phagocytosis by about 50%, whereas cells co-treated with RE and LPS exhibited a marked 37% increase in FITC-dextran uptake.

## 3. Discussion

In recent years, increasing attention has been paid to the valorization of marine resources, especially in the context of sustainability and the circular bioeconomy. The seagrass *P. oceanica* represents a rich source of phytotherapeutic compounds. Indeed, preliminary studies indicate that its extracts possess antioxidant, anti-inflammatory, antimicrobial, and anti-diabetic activities, suggesting that the species may represent a promising source of bioactive molecules for therapeutic and nutraceutical applications [24]. This research involved the preparation of GLE and RE from *P. oceanica* grown along the northern coast of Sicily, followed by a comparative evaluation of their potential modulatory impacts within a well-characterized inflammatory environment, modeled by LPS-primed RAW 264.7 macrophages. The information gathered indicates that these extracts show remarkable potential as potential anti-inflammatory and immunomodulatory agents.

Co-treatments of RAW 264.7 macrophages in the presence of either GLE or RE resulted in a reduction in NO release, associated with a down-regulation of *Nos2* expression, COX-2, and TNFα protein levels, and NF-κB activation. No effect was exerted on the release of IL-6. On the other hand, RE proved to exert a more significant anti-inflammatory influence compared to GLE. This included its capacity to lower IL-1β production, a factor known to amplify leukocyte responses [25] and a trait not shared by GLE, which induced a prominent up-regulation of the cytokine. Exposure to RE also led to a more robust increase in the anti-inflammatory and immunosuppressant cytokine IL-10 [26]. Furthermore, opposite to GLE, RE positively influenced endocytic function, a principal component of innate immunity and a significant gauge of macrophage activity [27]. As a result, the potent modulatory effect observed with RE appeared to be specific to particular roles of classically activated macrophages, notably, anti- and pro-inflammatory cytokine and iNOS expression, without affecting but rather stimulating their endocytic ability.

Co-treatment of RAW 264.7 macrophages with both extracts led to an elevation in pJNK and pAKT levels, with the pAKT increase being more pronounced in the presence of RE, alongside a reduction in pERK, with a more substantial decrease in the presence of GLE. Different from GLE and LPS, co-treatment of RAW 264.7 macrophages with RE and LPS drastically up-regulated the activation of p38 MAPK. Of note, various research findings, e.g., [28,29,30], have established a correlation between an increased phosphorylation level of p38 MAPK in macrophages and augmented phagocytic activity. Another report showed that ERK suppression, which we found was prominent in LPS/GLE co-treatment, attenuated macrophage phagocytosis [31]. These observations may be applicable to our research model, though further investigations are essential for validation.

Abruscato et al. [5] have previously characterized the polyphenols and proteins present in GLE and RE. The phenolic and protein component profiles of the two extracts are reported in Tables S1 and S2, respectively. Although a full elucidation of the precise biochemical pathways and possible synergisms by which the components of the extracts produce their effects is still required, data from the literature, summarized in Table 5, suggest that some of their polyphenolic constituents are possibly instrumental in the immunomodulatory and anti-inflammatory response reported here.

On the other hand, the selective presence or preferential enrichment of specific constituents within either extract may account for the diversified biological responses observed in LPS-stimulated RAW 264.7 cells co-treated with GLE or RE. The data presented in Table 4 suggest that the trace amounts of p-coumaric acid in GLE may be responsible for the down-regulation of the endocytic activity. Notably, Pragasam et al. [35] revealed a p-coumaric acid-attributable suppression in the phagocytic responsiveness of macrophages, as substantiated by carbon clearance tests in rats. This was ascribed to the stabilization of the cell membrane and an induced quiescent condition, leading to a reduction in macrophage functioning and, consequently, to a suppressive effect on this particular aspect of the immune response. Conversely, the endocytosis stimulated by RE could be ascribed to the selective presence of vanillic acid. Specifically, Zhu et al. [47] demonstrated that this substance can induce a pro-inflammatory M1-like phenotype in macrophages by activating the host stimulator of interferon genes (STING) signaling pathway, which resulted in a higher phagocytic rate.

Regarding the protein constituents within the extracts, a mass spectrometry-based proteomic assessment previously conducted on GLE and RE [5] revealed 100 identified proteins through bioinformatic similarity searches in multiple databases. A re-evaluation of these findings pinpointed some components potentially associated with the anti-inflammatory and immunomodulatory effects of the extracts, consistent with the literature showing the effects of their exogenous introduction into the culture medium of LPS-stimulated macrophages (Table 6).

Nonetheless, the potential involvement of trace proteins, which remained unidentified during proteomic analysis, or other water-soluble constituents, such as macro-, microelements, and carbohydrates [54,55], along with potential synergistic interactions among the existing compounds, cannot be dismissed. Moreover, the “active principle(s)” must retain stability throughout lyophilization, resuspension, and freeze–thawing cycles, which constitute the preparatory steps for the extracts prior to biological assays.

This study demonstrated that the two aqueous extracts from *P. oceanica’s* green leaves and rhizomes can modulate crucial inflammatory pathways in macrophages. This constitutes a significant advancement, providing evidence that reinforces the biological properties previously attributed to this species. While this research does not aim for immediate therapeutic application, it delivers fundamental knowledge indispensable for future investigations. Through the elucidation of how its extracts influence molecular targets, this study identified some processes by which *P. oceanica* can exert protective actions in inflammatory conditions. These findings provide a stronger scientific rationale for considering this endemic Mediterranean plant as a potential source for the discovery of new anti-inflammatory or immunomodulatory agents suitable for subsequent synthetic development. Overall, this work represents a preliminary but essential step for forthcoming studies, delineating the mechanisms by which bioactive compounds from *P. oceanica* regulate macrophages’ inflammatory reactions. Such insights are crucial for the isolation, synthesis, and in vivo deployment of these active constituents.

## 4. Materials and Methods

### 4.1. GLE and RE

GLE and RE were prepared following the protocol reported by Abruscato et al. [5]. Subsequent to storm events, which naturally detach and deposit *P. oceanica* plant material along the coastline, green leaves and rhizomes of this species were harvested from the Gulf of Palermo (Sicily, Italy). Each sample underwent a seawater rinse to remove sand and debris, was then placed into sterile bags, and subsequently transported to the laboratory within insulated cooler containers. Upon arrival at the laboratory, the samples were subjected to extensive washing, first with abundant tap water and subsequently with distilled water, thereby facilitating the removal of remaining sand particles, epiphytic material, and any residual salt component. After this cleaning step, the leaves and rhizomes underwent independent processing. The individual tissue types were flash-frozen, utilizing liquid nitrogen, and immediately pulverized, employing a mortar and pestle, resulting in a finely dispersed, uniform powder. This cryogenic grinding step was essential to prevent the degradation of thermolabile metabolites and proteins. Then, the resulting powders were reconstituted in a 2 M acetic acid solution supplemented with a protease inhibitor cocktail (1:200; P8340, Sigma, St. Louis, MO, USA) to reduce protein degradation during the extraction process. The complete dispersion of the plant material was ensured through homogenization using a T-25 digital Ultra-Turrax instrument (IKA, Staufen, Germany), after which the samples were subjected to sonication on ice with an ultrasonic processor (Sonics & Materials Inc., Danbury, CT, USA) to facilitate cellular disruption and enhance the release of soluble constituents. Following sonication, the samples were centrifuged at 15,500 rpm for 20 min at 4 °C to pellet the insoluble debris and collect the supernatants. The resulting supernatants were subsequently passed through sterile filters (pore size = 0.22 μm) to remove any residual particulates and ensure their sterility. The filtered extracts were frozen and lyophilized with an Alpha 2–4 LD plus instrument (Martin Christ, Osterode am Harz, Germany) to concentrate the soluble fraction and obtain a stable powder. Prior to experimentation, the lyophilized material was rehydrated with sterile distilled water and stored at –20 °C to safeguard its chemical characteristics. A comprehensive characterization of the polyphenolic composition and partial proteomic profiles of both GLE and RE, prepared following this procedure, has been previously reported in Abruscato et al. [5].

### 4.2. Cell Culture

The mouse macrophage cell line RAW 264.7 (RRID:CVCL_0493) was maintained in glutamine-containing Dulbecco’s Modified Eagle Medium (D-MEM, Sigma), supplemented with 10% fetal calf serum (Sigma) and antibiotics (100 U/mL penicillin and 100 μg/mL streptomycin; Sigma) at 37 °C in a 5% CO_2_ fully humidified atmosphere.

### 4.3. Viability Assay

For the viability test, cells in the exponential growth stage were seeded at a density of 5500/well in 96-well plates, allowed to settle overnight, and then grown either in control conditions or exposed to a range of concentrations of GLE or RE for 24 h. After adding 3-(4,5-dimethylthiazol-2-yl)-2,5 diphenyl tetrazolium bromide (MTT; Merck, Milan, Italy) and solubilizing the cells, the absorbance of the dissolved formazan was assessed using an automated microplate reader at a wavelength of 550 nm.

### 4.4. NO Production

As reported elsewhere [56], RAW264.7 cells were seeded at a density of 150,000/well in 24-well plates, allowed to settle overnight, and then cultured either under control conditions or treated with various concentrations of GLE (ranging from 0.25 to 20 μg/mL) or RE (ranging from 0.025 to 1 μg/mL) with or without the addition of 100 μL of LPS (final concentration: 0.1 μg/mL; O55:B5, Sigma) for 24 h. Following incubation with the Griess reagent (Biotium, Fremont, CA, USA), the absorbance of the culture media was quantified at 548 nm using an automated microplate reader. The NO release ratio in each experimental setup was determined by assessing the optical density of the media from the exposed cells relative to that of the control.

### 4.5. qRT-PCR

The cells utilized in the qRT-PCR analysis were grown in control conditions or underwent treatment with 100 μL of LPS (final concentration: 0.1 μg/mL) with or without addition of either 10 μg/mL GLE or 0.1 μg/mL RE for 24 h. Total RNA extraction was carried out using the PureLink RNA Mini kit (Thermo Fisher, Waltham, MA, USA) incorporating an on-column DNase treatment with the PureLink DNase set (Thermo Fisher), in accordance with the manufacturer’s protocol. Ten U of Transcriptor reverse transcriptase (Roche, Manheim, Germany) and random hexamer primers were used to synthesize the cDNAs, following the manufacturer’s instructions. The real-time qPCR analysis was executed using the Applied Biosystems 7500 Real-time PCR system (Applied Biosystems, Waltham, MA, USA) with the SYBR Green qPCR Master Mix (MedChem Express, Monmouth Junction, NJ, USA) and the specific primer sets listed in Table 7, as reported [5,8]. The amplification’s specificity underwent testing through real-time PCR melting analysis. The quantification of samples utilized the 2^−ΔΔCt^ method, with transcript levels normalized to that of GAPDH to compensate for any variation in the RNA input amount. Relative expression was quantitated by calculating the ratio of the normalized target gene value in each treated sample to the normalized value obtained from control samples.

### 4.6. Western Blot

The cells utilized in the Western blot analysis were grown in control conditions or underwent treatment with 100 μL of LPS (final concentration: 0.1 μg/mL) with or without addition of either 10 μg/mL GLE or 0.1 μg/mL RE for 24 h. As previously reported [5,8], RAW 264.7 cell cultures were subjected to harvesting and lysis in a buffer solution containing 7 M urea, 2% CHAPS, 10 mM DTT, and a protease inhibitor cocktail, all purchased from Sigma. Electrophoresis of protein samples was performed using 13% SDS-PAGE, followed by the transfer of proteins to nitrocellulose membranes. The membranes were incubated overnight at 4 °C with different rabbit primary antibodies, including anti-COX-2 (CQA2563 from Cohesion Biosciences, London, UK; diluted to 1:500), anti-ERK 1/2 (NB-22-2988 from NeoBiotech, Seoul, Republic of Korea, Nanterre, F; diluted to 1:500), anti-pERK (Tyr222/205) (NB-22-0657 from NeoBiotech; diluted to 1:1000), anti-IL-1β (FNab04209 from Fine Test, Wuhan, China; diluted to 1:1000), anti-IL-10 (CQA6564 from Cohesion Biosciences; diluted to 1:1000), anti-JNK (10023-1-AP from Thermo Fisher; diluted to 1:1000), anti-pJNK/SAPK1 (Thr183, Tyr185) (600-380 from Thermo Fisher; diluted to 1:1000), anti-NF-κB p65 (ab76311 from Abcam; diluted to 1:1000), anti-NF-kB p65 (Ser536) (BS-0928R from Bioss, Boston, MA, USA; diluted to 1:1000), anti-p38 (CPA5094 from Cohesion Biosciences; diluted to 1:750), anti-p38 (pT180/Y182) (CPA5055 from Cohesion Biosciences; diluted to 1:750), and, for internal control, anti-β-actin (NB-22-1460 from NeoBiotech; diluted to 1:1000). Following a 1 h-incubation at ambient temperature with the peroxidase-conjugated secondary antibody (ab6721 from Abcam; diluted to 1:3000), the immunoreactions were visualized using an enhanced chemiluminescence system (Versadoc MP Imaging System, Bio-Rad, Hercules, CA, USA) and the SuperSignal West Pico Plus substrate (Thermo Fisher). Signal intensity was quantitated using either Quantity One v.4.6.8 (Bio-Rad, Hercules, CA, USA) or ImageJ v.1.52a software, with normalization performed relative to the intensity of the β-actin band.

### 4.7. Enzyme Linked Immunosorbent Assay (ELISA)

The amounts of IL-6 and TNFα released in the cell media of control and treated macrophages were comparatively evaluated using the Mouse IL-6 ELISA kit (RAB0308-1KT, Sigma) and the Mouse Tumor Necrosis Factor α ELISA kit (RAB0477-1KT, Sigma), following the manufacturer’s instructions.

### 4.8. Endocytosis Assay

To quantify the effect of the co-treatments on the bulk-phase endocytic ability of the macrophages, RAW 264.7 cells were grown in control conditions or treated with LPS with or without addition of either 10 μg/mL GLE or 0.1 μg/mL RE for 24 h. Subsequently, the cells were incubated at 37 °C for 1 h with 100 μL of fluorescein isothiocyanate (FITC)-dextran (final concentration: 1 mg/mL; MW 40000; MedChem Express) and then subjected to flow cytometric analysis to evaluate their uptake, as described by Rod-In et al. [59]. Propidium iodide staining was performed in parallel to determine the effect of the treatment on cell death. Flow cytometry was conducted using a FACSCanto analyzer (BD Biosciences, Franklin Lakes, NJ, USA) evaluating 10,000 single-cell events. The analysis of the fcs files was performed using the web-based tool Floreada (https://floreada.io; accessed on 6 March 2025).

### 4.9. Statistics

Data are presented as mean ± s.e.m. One-way variance analysis (ANOVA) with the Holm–Sidak comparison procedure and normality test were performed. For statistical analysis, the SigmaPlot v.11.0 software (SYSTAT, San Jose, CA, USA) was used.

## 5. Conclusions

In conclusion, the comparative study involving GLE and RE has revealed that the water-soluble phytocomplexes obtained from the two distinct anatomical parts of *P. oceanica* possess macrophage-modulating capabilities exerting both immune-regulatory and anti-inflammatory actions in vitro, despite their specific bioactive capacities not being identical. These new attributes add up to the already-documented anticancer and anti-diabetic properties of the two biological matrices [5,8,19], which are noteworthy for their straightforward, economical, and methodologically uncomplicated extraction. The results reported here amplify the focus on the therapeutic potential of GLE and RE in the biomedical field, thereby prompting additional in vivo research to ascertain their impact on inflammatory and immune-compromised conditions.

## Figures and Tables

**Table 1:** Relative quantification of qRT-PCR for *Nos2* in RAW 264.7 cells treated with 0.1 μg/mL LPS or co-treated with 10 μg/mL GLE + 0.1 μg/mL LPS or 0.1 μg/mL RE + 0.1 μg/mL LPS for 24 h (treated/control ratio expressed as mean ± s.e.m. of triplicate experiments). One-way ANOVA followed by Holm–Sidak comparison procedure was used. ** *p* < 0.001 compared to LPS. Normality test vs. control passed.

LPS	GLE + LPS	RE + LPS
260.6 ± 4.3	0.85 ± 0.02 **	0.72 ± 0.01 **

**Table 2:** Relative quantification of qRT-PCR for *Ptgs2* in RAW 264.7 cells treated with 0.1 μg/mL LPS or co-treated with 10 μg/mL GLE + 0.1 μg/mL LPS or 0.1 μg/mL RE + 0.1 μg/mL LPS for 24 h (treated/control ratio expressed as mean ± s.e.m. of triplicate experiments). One-way ANOVA followed by Holm–Sidak comparison procedure was used. * *p* < 0.05 compared to LPS. Normality test vs. control passed.

LPS	GLE + LPS	RE + LPS
1.52 ± 0.03	0.84 ± 0.05 *	0.68 ± 0.04 *

**Table 3:** Relative quantification of qRT-PCR for *Il1b*, *Il6*, and *Il10* in RAW 264.7 cells treated with 0.1 μg/mL LPS or co-treated with 10 μg/mL GLE + 0.1 μg/mL LPS or 0.1 μg/mL RE + 0.1 μg/mL LPS for 24 h (treated/control ratio expressed as mean ± s.e.m. of triplicate experiments). One-way ANOVA followed by Holm–Sidak comparison procedure was used. * *p* < 0.05; ** *p* < 0.001 compared to LPS. Normality test vs. control passed.

	LPS	GLE + LPS	RE + LPS
*Il1b*	230.83 ± 11.1	387.65 ± 4.6 **	227.24 ± 8.3
*Il6*	2.63 ± 0.04	2.87 ± 0.05 *	3 ± 0.01 **
*Il10*	2.6 ± 0.09	2.7 ± 0.05	3.3 ± 0.09 **

**Table 4:** Relative quantification of qRT-PCR for *Nfkb1* in RAW 264.7 cells treated with 0.1 μg/mL LPS or co-treated with 10 μg/mL GLE + 0.1 μg/mL LPS or 0.1 μg/mL RE + 0.1 μg/mL LPS for 24 h (treated/control ratio expressed as mean ± s.e.m. of triplicate experiments). One-way ANOVA followed by Holm–Sidak comparison procedure was used. * *p* < 0.05 compared to LPS. Normality test vs. control passed.

LPS	GLE + LPS	RE + LPS
1.18 ± 0.02	1.04 ± 0.02 *	1.02 ± 0.01 *

**Table 5:** Biological properties of polyphenols present in GLE and/or RE. n.q. = not quantifiable.

Molecule	Extract/Amount (μg/g)	Bioactivity	Reference
Caffeic acid	GLE/n.q.	Decrease in NO production, down-regulation of iNOS and COX-2 mRNA expression and protein levels	[32]
Caffeic acid methyl ester	GLE/0.37	Decrease in NO production, down-regulation of iNOS and COX-2 mRNA expression and protein levels, inhibition of NF-κB activation, decrease in TNFα release	[33]
Catechin	GLE/n.q. RE/n.q.	Decrease in NO production, down-regulation of iNOS, TNFα and COX-2 mRNA expression and protein levels, inhibition of NF-κB mRNA expression, increase in IL-10 release	[34]
p-Coumaric acid	GLE/n.q.	Inhibition of COX-2, decrease in TNFα release, increase in IL-10 release, inhibition of phagocytosis	[35,36]
Ellagic acid	GLE/n.q.	Decrease in NO production, inhibition of TNFα production	[37,38]
Gallic acid	GLE/n.q. RE/n.q.	Down-regulation of iNOS, TNFα and IL-1β expression, inhibition of NF-κB activation	[39,40]
Kaempferol	GLE/n.q.	Down-regulation of iNOS and COX-2 expression, inhibition of NF-κB activation, decrease in TNFα release, increase in IL-10 release	[41]
Kaempferol 3-O-glucoside	RE/n.q.	Decrease in NO production, down-regulation of iNOS and COX-2 mRNA expression and protein levels, decrease in TNFα release, decrease in IL-1β release	[42]
Myricetin	RE/n.q.	Decrease in NO production, down-regulation of iNOS protein levels, decrease in TNFα release, decrease in IL-1β release, increase in IL-10 release	[43]
Procyanidin dimer B type isomer 2	GLE/n.q. RE/0.20	Inhibition of NF-κB activation, inhibition of ERK activation, decrease in TNFα release, decrease in IL-1β release	[44]
Quercetin 3-O-galactoside	GLE/n.q. RE/10.81	Decrease in NO production, down-regulation of iNOS expression, inhibition of NF-κB activation, decrease in TNFα release	[45]
Vanillic acid	RE/0.6	Decrease in NO production, down-regulation of COX-2 expression, inhibition of NF-κB activation, decrease in TNFα release, stimulation of phagocytosis	[46,47]

**Table 6:** Biological properties of proteins present in GLE and/or RE.

Accession Number/Protein Description	Extract/Amount	Documented Bioactivity (*Source*)	Reference
A0A0K9P699 Glutathione transferase	RE 2.01 × 10^5^	Decrease in IL-1β and TNFα release, increase in IL-10 release, inhibition of NF-κB and ERK activation (*Fasciola hepatica)*	[48]
A0A0K9P7A0 Glyceraldehyde-3-phosphate dehydrogenase	GLE 1.81 × 10^6^ RE 6.44 × 10^5^	Decrease in TNFα release, increase in IL-10 release, inhibition of phagocytosis (*rabbit muscle)*	[49]
A0A1D1YEH2 Heat shock cognate protein	RE 1.19 × 10^5^	Decrease in NO production, inhibition of iNOS and COX-2 expression, inhibition of NF-κB and ERK activation, decrease in TNFα release (*recombinant)*	[50]
A0A0K9NPM0 70 kDa Heat shock-related protein	GLE 1.11 × 10^5^ RE 4.19 × 10^4^	Decrease in NO production, decrease in TNFα release (*recombinant)*	[51,52]
A0A0K9Q3S1 Nucleoside-diphosphate kinase	GLE 3.87 × 10^4^ RE 2.31 × 10^5^	Decrease in TNFα release (*Trichuris suis)*	[53]
A0A0K9Q334 Triose-phosphate isomerase	GLE 1.27 × 10^5^ RE 3.65 × 10^5^	Decrease in TNFα release (*Trichuris suis)*	[53]

**Table 7:** Primers used in qRT-PCR analysis.

Gene (Primer)	Sequence (5′→3′)	Reference
*Il1b* (sense)	TGGAAAAGCGGTTTGTCTTC	[57]
*Il1b* (antisense)	TACCAGTTGGGGAACTCTGC	
*Il6* (sense)	GAGGATACCACTCCCAACAGACC	[57]
*Il6* (antisense)	AAGTGCATCATCGTTGTTCATACA	
*Il10* (sense)	GCTGGACAACATACTGCTAACC	[58]
*Il10* (antisense)	ATTTCCGATAAGGCTTGGCAA	
*Nfkb1* (sense)	GAAATTCCTGATCCAGACAAAAAC	[58]
*Nfkb1* (antisense)	ATCACTTCAATGGCCTCTGTGTAG	
*Nos2* (sense)	CAGGAGGAGAGAGATCCGATTTA	[57]
*Nos2* (antisense)	GCATTAGCATGGAAGCAAAGA	
*Ptgs2* (sense)	TGCATGTGGCTGTGGATGTCATCAA	[58]
*Ptgs2* (antisense)	CACTAAGACAGACCCGTCATCTCCA	
*Gapdh* (sense)	GGCCTTCCGTGTTCCTAC	[57]
*Gapdh* (antisense)	TGTCATCATATCTGGCAGGTT	

## Data Availability

The data presented in the current study are available from the corresponding author upon request.

## Supplementary Materials

The following supporting information can be downloaded at: https://www.mdpi.com/article/10.3390/molecules30244685/s1, Table S1: Phenolic component profiles of GLE and RE; Table S2: Complete protein profiles of GLE and RE; Figure S1: Images depicting the complete protein blots.
