# Supplementary material for: Anti-Inflammatory and Immunomodulatory Effects of Aqueous Extracts from Green Leaves and Rhizomes of Posidonia oceanica (L.) Delile on LPS-Stimulated RAW 264.7 Macrophages
Source: Molecules. 2025 Dec 7;30(24):4685. doi: 10.3390/molecules30244685 (PMC12735532; doi:10.3390/molecules30244685)
Supplement: Supplementary file 1 [file molecules-30-04685-s001.zip › molecules-4014410-supplementary.pdf]

Anti-inflammatory and immunomodulatory effects of aqueous extracts from green leaves and rhizomes of *Posidonia oceanica* (L.) Delile on LPS-stimulated RAW 246.7 macrophages

Giulia Abruscato, Daniela Ganci, Federica Bellistrì, Roberto Chiarelli, Manuela Mauro, Aiti Vizzini, Vincenzo Arizza, Mirella Vazzana and Claudio Luparello

Supplementary Materials

Table S1: Phenolic component profile of GLE and RE obtained through HPLC/MS analysis by Abruscato et al. [5]. n.q. = non quantifiable

Polyphenol	GLE (µg/g)	RE (µg/g)
Delphinidin-3-glucoside	n.q	11.52
Quercetin 3-O-galactoside	n.q	10.81
Procyanidin dimer B type isomer 2	n.q	0.20
Procyanidin dimer B type isomer 3	n.q	0.30
Pro-Cyanidin-Dimer-B	n.q	-
Cyanidin 3-O-glucoside	-	-
Vanillic acid	-	0.6
Gallic acid	n.q	n.q.
Kaempferol 3-O-glucoside	-	n.q.
Kaempferol	n.q	-
Kaempferol 7-O-hexuronide	-	n.q.
Procyanidin trimer B type	-	n.q.
Gallic acid ethyl ester	-	n.q.
Catechin	n.q	n.q.
Epicatechin	-	n.q.
Myricetin	-	n.q.
Peonidin 3-O-hexoside isomer	-	n.q.
Malvidin 3-O-pentoside	-	n.q.
Quercetin 3-O-hexuronide	-	n.q.
Quercetin 3-O-(6″-malonyl) hexoside	n.q	-
Resveratrol tetramer	-	n.q.
Caffeic acid methyl ester	0.37	-
Caffeic acid	n.q	-
p-Coumaric Acid	n.q.	-
Ellagic acid	n.q	-
p-Hydroxybenzoic acid	-	-
Ferulic acid	-	-
Myricetin	-	-
Myricetin 3-O-hexoside	-	-
Petunidin 3-O-(6″-acetyl) hexoside	-	-

Table S2: Complete protein profile of GLE and RE obtained through MS-based proteomic analysis by Abruscato et al. [5]

Accession number/Protein/Organism source	GLE	RE
O47254_POSOC Ribulose bisphosphate carboxylase large chain (Fragment) OS=Posidonia oceanica	1,81E+07	0
A0A843XPL2_COLES Mechanosensitive ion channel protein OS=Colocasia esculenta	7,32E+06	5,62E+06
A0A0K9Q0R4_ZOSMR phosphopyruvate hydratase OS=Zostera marina	5,81E+05	1,55E+07
A0A0K9Q5Y8_ZOSMR 5-methyltetrahydropteroyltriglutamate--homocysteine S-methyltransferase OS=Zostera marina	8,18E+05	5,92E+05
A0A0K9P7A0_ZOSMR Glyceraldehyde-3-phosphate dehydrogenase OS=Zostera marina	1,81E+06	6,44E+05
F1BXA2_WOLAR Calmodulin-related protein CAM53 OS=Wolffia arrhiza	1,25E+06	3,85E+06
A0A1D1Z792_9ARAE Polyubiquitin (Fragment) OS=Anthurium amnicola	3,46E+05	9,88E+05
A0A7I8IZJ3_SPIIN UTP--glucose-1-phosphate uridylyltransferase OS=Spirodela intermedia	3,01E+05	1,61E+05
A0A843VEE0_COLES Glyceraldehyde-3-phosphate dehydrogenase OS=Colocasia esculenta	1,15E+06	0
A0A0K9NYL5_ZOSMR ATP synthase subunit beta OS=Zostera marina	3,04E+05	5,07E+05
A0A1D1Z3T7_9ARAE Adenosylhomocysteinase (Fragment) OS=Anthurium amnicola	1,06E+05	6,39E+05
A0A843V8I2_COLES Lactoylglutathione lyase OS=Colocasia esculenta	7870	4,74E+05
A0A1D1ZEX7_9ARAE phosphoglycerate mutase (2,3-diphosphoglycerate-independent) OS=Anthurium amnicola	2,15E+05	5,31E+05
A0A1D1XUN0_9ARAE Superoxide dismutase OS=Anthurium amnicola	3,92E+05	1,95E+06
A0A1D1Z6U7_9ARAE Calmodulin OS=Anthurium amnicola	2,47E+05	1,95E+05
A0A1D1YR85_9ARAE 14-3-3-like protein C OS=Anthurium amnicola	2,16E+05	3,16E+05
A0A1D1YV12_9ARAE Actin-5C (Fragment) OS=Anthurium amnicola	2,74E+05	4,15E+05
A0A0K9Q334_ZOSMR Triose-phosphate isomerase OS=Zostera marina	1,27E+05	3,65E+05
A0A843W244_COLES Glyceraldehyde-3-phosphate dehydrogenase OS=Colocasia esculenta	6,91E+05	0
A0A0K9P9D6_ZOSMR Malate dehydrogenase OS=Zostera marina	6,28E+05	4,68E+05
A0A7I8IRM1_SPIIN 4-hydroxy-4-methyl-2-oxoglutarate aldolase OS=Spirodela intermedia	7330	6,38E+05
A0A0K9Q2C1_ZOSMR Photosystem II oxygen-evolving enhancer protein OS=Zostera marina	4,15E+05	0
A0A7I8JAP1_SPIIN Glutamate dehydrogenase OS=Spirodela intermedia	2,48E+04	7,47E+05
A0A0G3F6W2_9ARAE ATP synthase subunit beta, chloroplastic OS=Dieffenbachia seguine	4,30E+05	1,44E+04
A0A0K9Q207_ZOSMR Alcohol dehydrogenase 1 OS=Zostera marina	3,56E+04	4,60E+05
A0A0K9P2K2_ZOSMR Triose-phosphate isomerase OS=Zostera marina	1,22E+05	3,26E+05
A0A0G3F9P0_9ARAE ATP synthase subunit alpha, chloroplastic OS=Dieffenbachia seguine	2,14E+05	0
A0A1D1XJJ4_9ARAE Heat shock protein 81-2 (Fragment) OS=Anthurium amnicola	2,99E+05	2,58E+04
A0A1D1YHL8_9ARAE Sedoheptulose-1,7-bisphosphatase, chloroplastic OS=Anthurium amnicola	2,96E+05	7,75E+04
A0A7I8JCV7_SPIIN Pyruvate kinase OS=Spirodela intermedia	1,22E+05	7,75E+04
A0A1D1XTS3_9ARAE GTP-binding protein YPTM2 (Fragment) OS=Anthurium amnicola	0	2,35E+05
A0A0K9Q3S1_ZOSMR nucleoside-diphosphate kinase OS=Zostera marina	3,87E+04	2,31E+05
A0A0K9P7Q7_ZOSMR Peptidyl-prolyl cis-trans isomerase OS=Zostera marina	0	1,96E+05
A0A843WDB0_COLES ACB domain-containing protein OS=Colocasia esculenta	5,34E+04	3,72E+05
A0A1D1Z5E0_9ARAE hydroxymethylbilane synthase (Fragment) OS=Anthurium amnicola	2,57E+06	2,07E+07
A0A0K9NHA8_ZOSMR Peptidyl-prolyl cis-trans isomerase OS=Zostera marina	0	8,24E+04
A0A1D1YZX9_9ARAE 2-Cys peroxiredoxin BAS1-like, chloroplastic (Fragment) OS=Anthurium amnicola	5850	6,58E+04
A0A7I8JWW7_SPIIN Peroxidase OS=Spirodela intermedia	6,97E+04	2,63E+05
A0A1D1YK72_9ARAE transaldolase OS=Anthurium amnicola	2,28E+05	8,85E+04
A0A0K9PQ40_ZOSMR Ferredoxin--NADP reductase, chloroplastic OS=Zostera marina	1,37E+05	0

Table S2 (continued)

Accession number/Protein/Organism source	GLE	RE
A0A0K9PYW1_ZOSMR NADP-dependent glyceraldehyde-3-phosphate dehydrogenase OS=Zostera marina	5,56E+05	4,85E+05
A0A7I8JI83_SPIIN RuBisCO large subunit-binding protein subunit beta, chloroplastic OS=Spirodela intermedia	3,28E+05	0
A0A0K9NPD5_ZOSMR Calreticulin OS=Zostera marina	8,19E+04	9,28E+04
A0A0K9NPM0_ZOSMR Stromal 70 kDa heat shock-related protein, chloroplastic OS=Zostera marina	1,11E+05	4,19E+04
A0A0K9PHA0_ZOSMR Protein CutA 1, chloroplastic OS=Zostera marina	0	4,93E+05
A0A7I8KK42_SPIIN Lactoylglutathione lyase OS=Spirodela intermedia	0	9,00E+04
A0A843TVU3_COLES Malic enzyme OS=Colocasia esculenta	0	7,74E+04
A0A843W9S8_COLES Aspartate aminotransferase OS=Colocasia esculenta	4,20E+04	0
A0A843V931_COLES Histone H4 OS=Colocasia esculenta	5,03E+04	1,21E+05
A0A1B2ARS1_9LILI ATP synthase subunit alpha OS=Aponogeton distachyos	2,43E+04	3,71E+04
A0A7I8K3J0_SPIIN transketolase OS=Spirodela intermedia	6,67E+04	0
A0A0K9P881_ZOSMR Leucyl aminopeptidase OS=Zostera marina	9,56E+04	4,23E+04
A0A1D1ZBK1_9ARAE NAD(P)H dehydrogenase (quinone) OS=Anthurium amnicola	4,98E+04	1,28E+05
A0A0K9P513_ZOSMR Phosphoglycerate kinase OS=Zostera marina	1,06E+05	0
A0A7I8L9G2_SPIIN Ras-related protein RABH1b OS=Spirodela intermedia	0	1,33E+05
A0A0K9PJK1_ZOSMR Thiamine thiazole synthase, chloroplastic OS=Zostera marina	1,19E+05	1,00E+05
A0A0K9PFX9_ZOSMR Chaperonin CPN60-2, mitochondrial OS=Zostera marina	0	3,16E+05
A0A1D1Z2Q2_9ARAE Proteasome subunit alpha type (Fragment) OS=Anthurium amnicola	0	1,79E+05
A0A1D1ZBQ7_9ARAE Glucose-1-phosphate adenylyltransferase (Fragment) OS=Anthurium amnicola	0	8,01E+04
A0A0K9NZB2_ZOSMR Proteasome subunit alpha type OS=Zostera marina	1,89E+05	8,92E+04
A0A1D1ZEX8_9ARAE methionine S-methyltransferase (Fragment) OS=Anthurium amnicola	1,15E+05	0
F8U875_AMOKO phosphoglucomutase (alpha-D-glucose-1,6-bisphosphate-dependent) OS=Amorphophallus konjac	0	1,22E+05
A0A0K9PU13_ZOSMR Isocitrate dehydrogenase [NADP] OS=Zostera marina	1,99E+04	2,07E+05
A0A1D1Z1S7_9ARAE RING-type domain-containing protein (Fragment) OS=Anthurium amnicola	1,07E+06	0
A0A0G3F797_9ARAE Photosystem II reaction center protein L OS=Dieffenbachia seguine	3,10E+05	0
A0A1D1YQU6_9ARAE Adenosylhomocysteinase (Fragment) OS=Anthurium amnicola	0	2,65E+05
Q9ATC0_ZANAE Ribulose bisphosphate carboxylase/oxygenase activase, chloroplastic (Fragment) OS=Zantedeschia aethiopica	1,90E+05	0
A0A1D1XR46_9ARAE Peroxidase (Fragment) OS=Anthurium amnicola	0	1,40E+05
A0A0K9PU82_ZOSMR Ferritin OS=Zostera marina	0	2,27E+05
A0A843UI87_COLES Aldo_ket_red domain-containing protein OS=Colocasia esculenta	0	7,13E+04
A0A1D1ZGY1_9ARAE Glutathione reductase OS=Anthurium amnicola	0	1,31E+05
A0A0K9NZL7_ZOSMR Proteasome subunit alpha type OS=Zostera marina	4,28E+04	5,16E+04
A0A0K9P699_ZOSMR glutathione transferase OS=Zostera marina	0	2,01E+05
A0A1D1YLJ7_9ARAE Phosphoglycerate kinase OS=Anthurium amnicola	6,99E+04	5,59E+04
A0A0K9P894_ZOSMR Malate dehydrogenase OS=Zostera marina	1,06E+05	0
A0A843WQL4_COLES transketolase OS=Colocasia esculenta	1,34E+05	0
A0A0K9NP87_ZOSMR Proteasome subunit alpha type OS=Zostera marina	2,53E+04	2,89E+04
A0A0K9P1R8_ZOSMR Serine hydroxymethyltransferase OS=Zostera marina	8,23E+04	0
HBB_HUMAN|(Common contaminant protein)	0	1,06E+05
A0A1D1Y526_9ARAE Ferritin OS=Anthurium amnicola	0	1,22E+05

Table S2 (continued)

Accession number/Protein/Organism source	GLE	RE
D2KL31_WOLAR Glutaredoxin-dependent peroxiredoxin OS=Wolffia arrhiza	1,32E+05	4,38E+04
A0A0K9PRU3_ZOSMR Cysteine synthase OS=Zostera marina	8,59E+04	8,89E+04
A0A1D1YEH2_9ARAE Heat shock cognate protein (Fragment) OS=Anthurium amnicola	0	1,19E+05
A0A0K9P8G2_ZOSMR Proteasome subunit alpha type OS=Zostera marina	3,17E+04	2,03E+05
A0A1D1XNG3_9ARAE Proteasome subunit alpha type (Fragment) OS=Anthurium amnicola	1,87E+04	5,08E+04
A0A1D1ZBB9_9ARAE Adenosine kinase (Fragment) OS=Anthurium amnicola	4,68E+04	3,92E+04
A0A1D1Z3I9_9ARAE isocitrate dehydrogenase (NADP(+)) (Fragment) OS=Anthurium amnicola	1,99E+04	6,51E+04
A0A7I8IGX1_SPIIN Nucleoside diphosphate kinase OS=Spirodela intermedia	4,69E+04	1,90E+04
A0A1D1XKQ1_9ARAE NADP-dependent D-sorbitol-6-phosphate dehydrogenase (Fragment) OS=Anthurium amnicola	0	1,45E+05
A0A7I8I9Q7_SPIIN Cysteine synthase OS=Spirodela intermedia	2,58E+04	8,85E+04
A0A0K9NRD5_ZOSMR coproporphyrinogen oxidase OS=Zostera marina	1,67E+05	0
A0A7I8J3Q6_SPIIN ATP-dependent Clp protease ATP-binding subunit OS=Spirodela intermedia	0	1,58E+05
A0A0K9PL60_ZOSMR Bifunctional aspartate aminotransferase and glutamate/aspartate-prephenateaminotransferase OS=Zostera marina	1,32E+05	0
A0A0K9PU80_ZOSMR Glutamine synthetase OS=Zostera marina	8,75E+04	0
A0A0K9PFW2_ZOSMR Vacuolar proton pump subunit B OS=Zostera marina	0	1,06E+05
A0A1D1ZGC8_9ARAE Proteasome subunit beta (Fragment) OS=Anthurium amnicola	1,40E+05	0
A0A0K9PU68_ZOSMR Proteasome subunit beta OS=Zostera	4,97E+04	7,69E+04
A0A1D1YE28_9ARAE Cell division cycle protein 48 (Fragment) OS=Anthurium amnicola	7,96E+04	0
A0A4Y1KCE6_9LILI Photosystem II D2 protein OS=Thalassia hemprichii	6,22E+04	0
A0A0K9NY84_ZOSMR Cysteine synthase OS=Zostera marina	1,43E+05	0

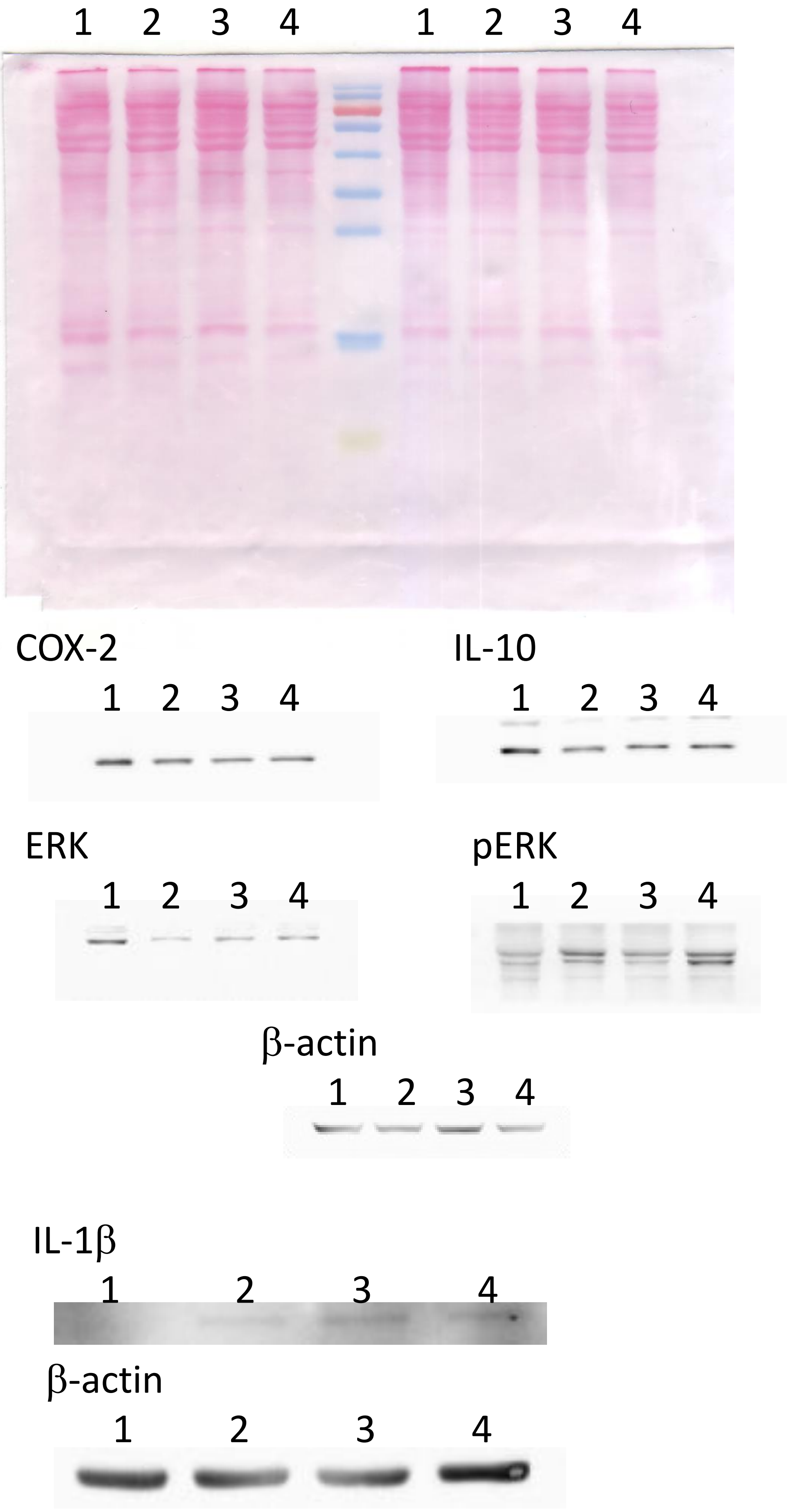
1 2 3 4 1 2 3 4
COX-2
1 2 3 4
IL-10
1 2 3 4
ERK
1 2 3 4
pERK
1 2 3 4
β-actin
1 2 3 4
IL-1β
1 2 3 4
β-actin
1 2 3 4

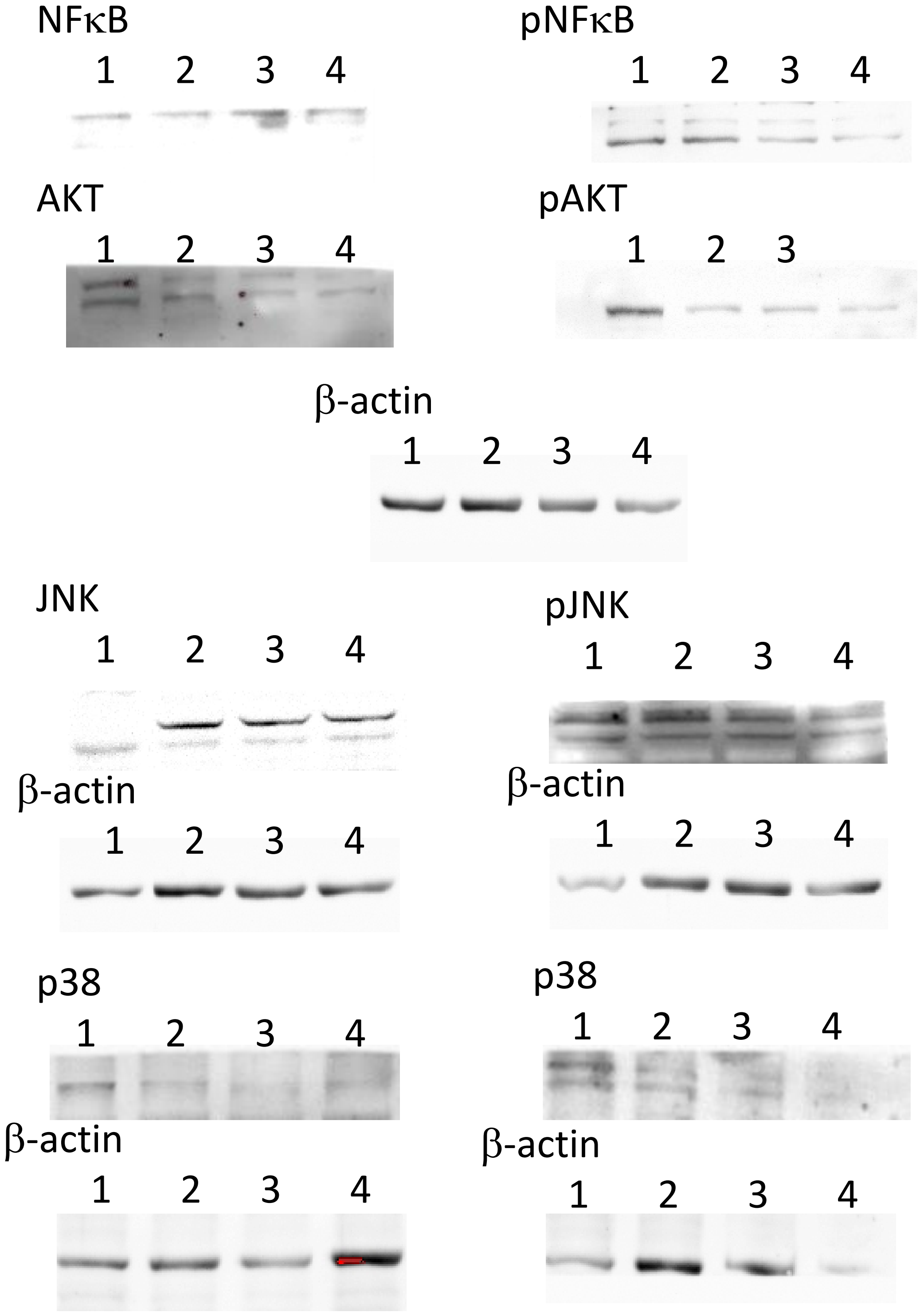

Figure S1: Representative image of the whole protein blot stained with Ponceau S and showing the molecular weight marker and images of the whole immunoblots whose trimmed inserts have been used for figures 4-7. Control = sample 1, LPS = sample 2, LPS + GLE = sample 3, LPS + RE = sample 4.
